# Associations Among Parental Caregiving Quality, Cannabinoid Receptor 1 Expression-Based Polygenic Scores, and Infant-Parent Attachment: Evidence for Differential Genetic Susceptibility?

**DOI:** 10.3389/fnins.2021.704392

**Published:** 2021-07-27

**Authors:** Amelia Potter-Dickey, Nicole Letourneau, Patricia P. Silveira, Henry Ntanda, Gerald F. Giesbrecht, Martha Hart, Sarah Dewell, A. P. Jason de Koning

**Affiliations:** ^1^Faculty of Nursing, University of Calgary, Calgary, AB, Canada; ^2^Owerko Centre, Child Development Centre, Department of Pediatrics, Cumming School of Medicine, Alberta Children’s Hospital Research Institute, University of Calgary, Calgary, AB, Canada; ^3^Department Community Health Sciences, Cumming School of Medicine, University of Calgary, Calgary, AB, Canada; ^4^Department of Psychiatry, Cumming School of Medicine, University of Calgary, Calgary, AB, Canada; ^5^Ludmer Centre for Neuroinformatics and Mental Health, Douglas Mental Health University Institute, McGill University, Montreal, QC, Canada; ^6^Department of Psychiatry, Faculty of Medicine, McGill University, Montreal, QC, Canada; ^7^School of Nursing, University of Northern British Columbia, Prince George, BC, Canada; ^8^Department of Biochemistry & Molecular Biology, University of Calgary, Calgary, AB, Canada; ^9^Department of Medical Genetics, University of Calgary, Calgary, AB, Canada

**Keywords:** expression-based polygenic risk score (ePRS), cannabinoid receptor gene 1 (CNR1), parent–child relationship quality, CARE-index, strange situation procedure, APrON study, attachment security, attachment disorganization

## Abstract

Attachment is a biological evolutionary system contributing to infant survival. When primary caregivers/parents are sensitive and responsive to their infants’ needs, infants develop a sense of security. Secure infant attachment has been linked to healthy brain and organ-system development. Belsky and colleagues proposed the term differential susceptibility to describe context-dependent associations between genetic variations and behavioral outcomes as a function of parenting environments. Variations in the Cannabinoid Receptor Gene 1 (CNR1) are associated with memory, mood, and reward and connote differential susceptibility to more and less optimal parental caregiving quality in predicting children’s behavioral problems.

**Aim:**

To determine if parental caregiving quality interacts with children’s expression-based polygenic risk score (ePRS) for the CNR1 gene networks in the prefrontal cortex, striatum, and hippocampus in predicting the probability of attachment security and disorganized attachment.

**Design:**

Prospective correlational methods examined maternal-infant pairs (*n* = 142) from which infants provided DNA samples at 3 months. Parental caregiving quality was assessed via the Child Adult Relationship Experiment (CARE)-index at 6 months, and attachment security via the Strange Situation Procedure at a mean age of 22 months. The CNR1 ePRSs include genes co-expressed with the CNR1 genes in the prefrontal cortex, striatum, or hippocampus, and were calculated using the effect size of the association between the individual single nucleotide polymorphisms from those genes and region-specific gene expression (GTEx). Logistic regression was employed (alpha < 0.05, two-tailed) to examine the main and interaction effects between parental caregiving quality and ePRSs in predicting attachment patterns. Interpretation of results was aided by analyses that distinguished between differential susceptibility and diathesis-stress.

**Results:**

Significant interactions were observed between (1) maternal sensitivity and ePRS in the striatum in predicting attachment security, (2) maternal unresponsiveness with the ePRS in the hippocampus in predicting disorganization, and (3) maternal controlling with the ePRS in the hippocampus in predicting disorganization.

**Conclusion:**

These findings offer support for genetic differential susceptibility to the quality of maternal sensitivity in the context of the ePRS in the striatum. However, the significant interactions between hippocampal ePRS and maternal unresponsiveness and controlling in predicting the probability of disorganization were more suggestive of the diathesis-stress model.

## Introduction

Since psychiatrist John Bowlby first considered the importance of infants’ secure attachments with their caregivers to later mental health, research on attachment patterns has exploded (Sroufe et al., 2005; Cassidy, 2016). Attachment theory has not only provided a basis for international research programs but has also become an influential perspective on child development in clinical and welfare practice (Sroufe et al., 2005; Kozlowska and Elliott, 2014; Teti and Kim, 2014). The most fundamental aspect of attachment theory is that a child’s attachment behavior has social-biological underpinnings promoting a vulnerable infant’s proximity to the attachment figure, improving their chance of survival (Simpson, 1999; Cassidy, 2016). When primary caregivers/parents are available and responsive to their infants’ needs, infants develop a sense of security, making them feel safe, secure, and protected (Bowlby, 1982; Benoit, 2004; Solomon and George, 2016). Infants anticipate their parents’ responses to their distress and shape their attachment behaviors accordingly (Benoit, 2004). When observed and scored, infant attachment behavior typically is classified into one of four attachment patterns: secure, insecure-avoidant, insecure-resistant, and disorganized (Ainsworth et al., 1978; Main and Solomon, 1986). A growing body of evidence links infant secure attachment patterns to healthy brain and organ-system development and insecure and disorganized attachment to increased levels of all-cause morbidity, chronic inflammation, coronary artery disease, and an array of mental health disorders (Schore, 2000, 2001; Sroufe, 2005; Puig et al., 2013).

Parental caregiving quality, typically characterized by qualities of maternal sensitivity, control, and responsiveness, predicts infants’ attachment pattern (De Wolff and van Ijzendoorn, 1997; van Ijzendoorn et al., 1999; Madigan et al., 2006; Crittenden, 2010; Bailey et al., 2017). Sensitivity is a caregiver’s ability to perceive, accurately interpret, and respond promptly and accurately to an infant’s cues (Ainsworth et al., 1978). High maternal sensitivity involves responding to infant/child cues that signal needs or distress, such as fussiness due to hunger or fatigue, in a timely fashion, while low maternal sensitivity is indicated by low responsiveness (Barnard and Guralnick, 1997). High maternal sensitivity also denotes behaviors contrary to overtly or covertly hostile behaviors or attempts to excessively control infant behavior in routine interactions (Kelly et al., 2008). While sensitive caregiving may support the development of acceptable emotional expressions and optimal regulation, harsh, controlling caregiving behaviors may undermine children’s emotional development. Therefore, high-quality parental caregiving is typically characterized by sensitive and responsive interactions attentive to infant needs while mitigating excessive intrusion and control. A greater degree of sensitivity shows the infant that the caregiver is dependable, which creates a secure base for the child then to explore the world (Thompson, 2016). Parental sensitivity is regarded as one of the most important determinants of infant attachment security (Fearon et al., 2006; Bakermans-Kranenburg and van Ijzendoorn, 2007; Colmer et al., 2011), while traumatic events thought to undermine parental caregiving predict disorganized attachment (Lyons-Ruth, 2015).

However, despite being an important factor in predicting attachment patterns, parental caregiving quality does not explain as much variance as one might expect (De Wolff and van Ijzendoorn, 1997; van Ijzendoorn et al., 1999; Bailey et al., 2017). There has been evidence to support associations between attachment patterns and several sociodemographic factors such as maternal age (Esma et al., 2018), socioeconomic status (Acevedo et al., 2012), migration background (Keller, 2018), infant sex (Weinberg et al., 1999; David and Lyons-Ruth, 2005), infant gestational age (Wille, 1991), descriptive factors such as maternal depression (Kohlhoff and Barnett, 2013), social support (Jacobson and Frye, 1991), and infant birth weight (Wille, 1991; Weiss et al., 2000). In addition, there is a growing body of evidence suggesting that individuals’ genetics may influence attachment patterns (Lakatos et al., 2000, 2002; Belsky and Beaver, 2011; Luijk et al., 2011; Belsky et al., 2015; Pappa et al., 2015; Golds et al., 2020). Specifically, disorganized attachment patterns have been linked to genetic variations of the genes responsible for regulating dopamine (DA; Lakatos et al., 2000, 2002; Gervai et al., 2005, 2007; van Ijzendoorn and Bakermans-Kranenburg, 2006).

The majority of the literature examining the roles of parental caregiving behavior and genetics in predicting attachment relates to neurotransmitters, particularly those implicated in reward processing (Bakermans-Kranenburg and van Ijzendoorn, 2016; Feldman, 2017). DA is a neurotransmitter associated with motivation or pleasure necessary to promote a response to environmental cues that signal reward and depend on carrying out a specific action or behavior to receive it (Du Hoffmann and Nicola, 2014). The endocannabinoid system (ECS) is implicated in a wide variety of brain functions, such as reward processing as well as memory, mood, and motor control. The Type 1 Cannabinoid Receptor (CB1), encoded by the Cannabinoid Receptor 1 (CNR1) gene, is a key component of the endocannabinoid system and is expressed in both the central and peripheral nervous systems, particularly on axon terminals in the cerebellum, hippocampus, basal ganglia, frontal cortex, amygdala, hypothalamus, and midbrain (Romero-Fernandez et al., 2013; Brzosko et al., 2015). The CB1 receptor is an important component of the ECS in the nervous system, regulating synaptic transmission by modulating neurotransmitters’ release, including DA (Tao et al., 2020). Two of the most commonly studied CNR1 polymorphisms include rs1049353 (Agrawal et al., 2012) and rs7766029 (Juhasz et al., 2009) in relation to different phenotypic outcomes, especially in the rs1049353 genotype, A allele. Previous studies support the notion that outcomes can vary with the different polymorphic variants of these genes. For example, when the CNR1-A allele is absent, the caregiving environment’s impact on children’s externalizing behaviors is attenuated. Higher levels of negative caregiver control, in the presence of the CNR1-A gene, predicted parent-report of more externalizing behaviors in children. In comparison, lower levels of negative caregiver control predicted the report of less externalizing behaviors in a differentially susceptible manner (Letourneau et al., 2019).

Genetic variations can have varying functional effects in different biological contexts; thus, specific genes may produce different observable outcomes in response to either stressful or protective environments (Del Giudice, 2016). Belsky and Pluess (2009a) proposed utilizing the term differential susceptibility when describing genes associated with both adaptive and maladaptive changes in phenotypes in response to “supportive” and “unsupportive” parental caregiving environments. Parental caregiving quality incorporates constructs such as nurturing, acceptance, and cohesion, and involves behaviors toward the child (e.g., praising, encouraging, and giving physical affection), which signal to the child love, support, and acceptance (Barnes et al., 2000). In short, genetic differential susceptibility theory may explain why some infants appear to have increased susceptibilities to parental caregiving qualities. Genetic variation leading to neurobiological and temperamental traits characterized by highly sensitive and responsive stress physiology may determine increased susceptibility to stress and adversity (Del Giudice, 2016). Highly genetically susceptible children have disproportionately high morbidity rates when raised in adverse stressful environments; in addition, children with a higher degree of genetic susceptibility more frequently exhibit mental health symptoms in adolescence (Essex et al., 2011), exhibit epigenetic modifications (i.e., decreased DNA methylation; Goodman et al., 2018), and are more likely to exhibit behavioral problems under circumstances of low caregiver support (Skowron et al., 2014; Letourneau et al., 2019). In contrast, children with a high degree of genetic susceptibility become more socially integrated, have the lowest levels of illness (Boyce et al., 1995), and highest school engagement levels when receiving high-quality parental caregiving (Obradović et al., 2010). This dichotomy in children with a high degree of genetic susceptibility suggests a unique opportunity to identify individuals who could be at risk for poor health outcomes by assessing children’s genetic differential susceptibility to parental caregiving quality. However, a rival explanation for some of these associations is diathesis-stress, in which poor developmental experiences (e.g., low-quality parenting) are most likely to impact the development of individuals who carry vulnerability factors that result in maladaptation. Ascertaining whether parenting interacts with genetic factors in either a differential susceptibility or diathesis-stress manner is a subject of ongoing exploration (Garmezy et al., 1984; Roisman et al., 2012; Portella et al., 2020).

Novel genomic metrics that either predict gene expression in tissue-specific regions or use gene co-expression information may provide a more comprehensive view of a specific gene or a gene network’s role in modulating an individual’s response to environmental variations, compared to that provided by the single candidate gene approach (Gamazon et al., 2015; Barth et al., 2020). Expression-based polygenic risk scores (ePRS) offer one such approach to understand the underlying genetic background linked to behavioral outcomes (Hari Dass et al., 2019). ePRS is a genomic risk profiling method that recognizes a gene network contribution to a particular condition or outcome derived from a combination of small effects from many genetic variants. ePRS scores are derived based on transcriptional co-expression profiles from specific regions of the mouse (GeneNetwork) and human (Brainspan) brains, used to identify Single Nucleotide Polymorphisms (SNPs) functionally associated with gene expression in the human brain (GTEx). ePRS analyses provide a new paradigm to identify gene-by-environment interactions (McGrath et al., 2013; Plomin, 2013; Iyegbe et al., 2014; Silveira et al., 2017; Belsky et al., 2019; De Lima et al., 2020).

When attachments form in early infancy, activation and closer links are observed among neurobiological brain systems underpinning affiliation, reward, and stress management (Ulmer-Yaniv et al., 2016). Functional magnetic resonance imaging (fMRI) has been used to investigate the brain activity associated with humans’ various social attachments (Feldman, 2017). These fMRIs provide evidence for three main inter-connected neural systems that integrate to establish, maintain, and enhance our attachments to others, including the reward-motivation system (Berridge and Robinson, 1998), the embodied simulation/empathy network (Gallese, 2014), and mentalizing processes (Frith and Frith, 2006). The reward-motivation system comprises the striatum (nucleus accumbens, caudate, and putamen), amygdala, ventral tegmental area, orbitofrontal cortex, ventromedial prefrontal cortex, and anterior cingulate cortex (ACC). The existence of convergent projections from the cortex to the striatum, along with hippocampal and amygdala-striatal projections, places the striatum as a central entry port for processing emotional/motivational information supporting human attachment (Haber and Knutson, 2010; Robinson et al., 2012; Pauli et al., 2016). The reward-motivation system employs DA and oxytocin rich pathways (Schultz, 2000; Berridge et al., 2009; Haber and Knutson, 2010) and supports multiple attachment-related motivational behaviors, such as social orienting, social seeking, and maintaining contact (Acevedo et al., 2012; Chevallier et al., 2012). Attachments have an intrinsic motivational value that combine immediate hedonic responses with approach motivation, goal-directed behavior, and learning (Berridge and Robinson, 1998).

The embodied simulation/empathy network includes the insula, ACC, inferior frontal gyrus, inferior parietal lobule, and supplementary motor area. Embodied simulation is an ancient evolutionary mechanism essential to grounding a ‘shared world’ in the brain and underpins the human capacity to build and maintain attachments (Craig, 2009; Gallese, 2014). Finally, the formation and maintenance of attachment bonds also rely on higher-order mentalizing processes that involve complex top-down inferences (Frith and Frith, 2006; Van Overwalle, 2009). Mentalizing processes underpin attachment and reinforce attachment formation by building on the individual’s ability to appreciate multiple perspectives, understand others’ goals and motives, and keep in mind their values and concerns (Ciaramidaro et al., 2014; Hari et al., 2015). The mentalizing system consists of frontotemporal–parietal structures, particularly the superior temporal sulcus, posterior cingulated cortex, temporoparietal junction, temporal pole, and medial prefrontal cortex (Feldman, 2017).

To the best of our knowledge, this is the first study that seeks to investigate if infant genetic susceptibility interacts with the quality of parental caregiving in predicting attachment patterns using observational measures. This understanding could offer empirical evidence of infants’ physiological responsivity to positive (and negative) parental caregiving (Barth et al., 2020). We propose utilizing the innovative approach of ePRS to determine if parental caregiving quality (i.e., sensitivity, unresponsiveness, and controlling) interacts with children’s ePRS for the prefrontal cortex, striatum, and hippocampus CNR1 gene networks in predicting the probability of secure and/or disorganized attachment. Previous studies examining various polymorphic variants, including CNR1, in relation to children’s behavior have suggested that they have the potential to interact with environmental influences in a differentially susceptible manner (Young et al., 2002; Letourneau et al., 2019). Due to the activation of the neurobiological systems associated with the ECS that underpin affiliation, reward, stress management, responsiveness to the environment, and mood (Lupica et al., 2004; Ranganathan and D’Souza, 2006; Hill et al., 2009; Zuurman et al., 2009; Zanettini et al., 2011; Feldman, 2017), and thus potential to relate to attachment pattern formation in infancy (Berridge and Robinson, 1998; Acevedo et al., 2012; Chevallier et al., 2012), we chose this specific gene (CNR1) and tissue-specific networks for study. We focused on the prefrontal cortex due to its association with cognitive, emotional functions, impulse control, and adaptive behaviors (Morecraft and Yeterian, 2002; Bechara and Van Der Linden, 2005), and the striatum for its involvement in the reward motivation system and potential to relate to attachment formation in infancy specifically (Feldman, 2017). Convergent projections from the cortex to the striatum, along with hippocampal and amygdala-striatal projections, places the striatum as a central entry port for processing emotional/motivational information supporting human attachments (Haber and Knutson, 2010; Robinson et al., 2012; Pauli et al., 2016; Feldman, 2017). Finally, as part of the limbic system, the hippocampus was chosen for its spatial and emotional memory involvement. The hippocampus plays an essential role in social memory and consolidating declarative or explicit memories of facts or events that enable conscious recall from long-term memory (Campbell and Macqueen, 2004). The ability to recognize and memorize familiar conspecifics (social memory) is a critical aspect of social interactions in animals (McGraw and Young, 2010; Okuyama et al., 2014, 2016). As the hippocampus develops, the infant can recognize and remember their caregiver and begin to feel a sense of pleasure with them during engaging interactions (Chambers, 2017).

We hypothesize that within the three selected brain regions (i.e., prefrontal cortex, striatum, and hippocampus): (1) higher maternal sensitivity will interact with ePRS for the CNR1 gene networks in predicting a higher probability of secure attachment and reduced probability of disorganization, (2) higher maternal controlling will interact with ePRS for CNR1 gene networks in predicting a reduced probability of secure attachment and higher probability of disorganization, and (3) higher maternal unresponsiveness will interact with ePRS for CNR1 gene networks in predicting a reduced probability of secure attachment and higher probability of disorganization.

## Materials and Methods

This secondary analysis employs data from the Fetal Programming Study (Giesbrecht et al., 2017), a sub-study derived from the larger Alberta Pregnancy Outcomes and Nutrition (APrON) longitudinal cohort study (Kaplan et al., 2014), which ended enrollment in 2012. The Fetal Programming Study aimed to examine biomarkers of maternal stress during pregnancy and collect data on parent–infant interaction quality and attachment (Kaplan et al., 2014; Giesbrecht et al., 2017; Letourneau et al., 2017). Ethics approval was obtained from the Conjoint Health Research Board at the University of Calgary in Alberta, Canada. All participants in the study completed a process of informed consent prior to participating. For this project’s scope, relevant data were collected at study visits during pregnancy and 3, 6, and 22 months postpartum.

### Participants and Recruitment

Recruitment of 294 pregnant women into the Fetal Programming study took place between 2011 and 2012 in a large western Canadian city. Expectant mothers were recruited through media advertisements and maternity, ultrasound, family medicine, and obstetric clinics (Kaplan et al., 2014). To be eligible at enrollment, mothers: (1) were less than 22 weeks pregnant, (2) were 16 years of age or older, (3) were pregnant with a singleton, (4) reported abstaining from alcohol and tobacco during pregnancy, (5) reported not receiving a glucocorticoid medication during pregnancy, and (6) reported no known fetal complications. Mothers were excluded if they could not answer questions in English or planned to move out of the region during the study’s timeframe (Kaplan et al., 2014). Of the 294 recruited participants in the Fetal Programming Study, 142 maternal infant-pairs provided an infant Deoxyribonucleic Acid (DNA) sample in the form of a buccal swab or blood sample with sufficient quantity to calculate ePRS and completed all assessments of maternal-infant relationship quality and attachment patterns (Thomas et al., 2017).

### Procedures and Measures

Data were collected on mothers’ demographic characteristics at enrollment and infant demographic characteristics at birth. Additional data were collected during pregnancy and postpartum on depression and social support. Blood was drawn, or buccal cells were collected from children at 3 months of age. Observational assessments of maternal-infant interaction quality (predictor) were conducted at 6 months of age and infant attachment pattern (outcome) at 22 months.

#### Predictors

To measure *parental caregiving quality*, we employed the Child Adult Relationship Experiment (CARE)-Index (Crittenden, 2010). It is valid with infants from birth up to 15 months (Crittenden and Bonvillian, 1984; Crittenden and DiLalla, 1988; Ward and Carlson, 1995; Leadbeater et al., 1996; Leventhal et al., 2004), and inter-rater reliability values range between *r* = 0.73 and 0.95 (Leventhal et al., 2004; Azar et al., 2007). When the infants were 6 months of age, a 5-min observational procedure was carried out by videotaping the mother–infant pairs engaging in play with age-appropriate toys. Seven aspects of interaction behavior are assessed, including facial expression, verbal expression, positional and body contact, affection, turn-taking, control, and activity choice. Total scores for parental sensitivity, controlling, and unresponsiveness are derived, ranging from 0–14 (Crittenden, 2010). Author Letourneau is a reliable CARE-Index coder and supervised the administration and blinded data coding. Trained, independent designates coded video recordings at Crittenden’s laboratory, who achieved a 94.4% inter-rater agreement on the three observable constructs. For each of the CARE-Index subscales (i.e., sensitivity, controlling, and unresponsiveness), three scoring category groups were created, including: “low” which included maternal-child dyads that scored less than one standard deviation below the calculated mean; “mean,” which included maternal-child dyads that scored within one standard deviation above or below the calculated mean; and “high” which included maternal-child dyads that scored more than one standard deviation above the calculated mean. These categories enabled data in graphs and figures to be interpreted more readily.

To collect *DNA for analysis*, blood was drawn from infants at a study visit at 3 months of age. All samples were drawn by a certified phlebotomist using either a butterfly needle or a 25-gauge 3/4 inch infant needle. The blood samples were processed within 6 h of collection at the affiliated hospital genetics laboratory. This process involved spinning the vacutainer at 3,000 rotations per minute for 15 min to separate the plasma, buffy coat (i.e., leukocytes and platelets), and erythrocytes. The buffy coat was extracted using a pipette from the collection container, placed into a microcentrifuge tube, and stored at −80°C for DNA extraction at a later date. Buccal epithelial cells (BEC) were also collected from infants if their blood draw yield was low or unobtainable. This was done by rubbing a sterile cytology brush up and down the infant’s entire cheek ten times on two different swabs to ensure an adequate sample was obtained. The BEC and processed blood leukocytes were kept in short-term storage at −80°C before DNA extraction. DNA extraction was done by cell lysis, followed by purification using the Gentra Puregene method (Qiagen, Venlo, Limburg, Netherlands). The samples were processed for DNA purification using the Autopure method (Qiagen, Venlo, Limburg, Netherlands) and processed further using the cell lysate program. Samples were left open to air allowing for evaporation of excess ethanol, and low-TE buffer was added to the tubes. After DNA extraction, the isolated DNA samples were stored at 4°C at the affiliated hospital genetics laboratory.

The genetic data were extracted using Illumina HumanCoreExome BeadChipVersion 1 and subjected to quality control (QC) procedure using PLINK 1.9 (Chang et al., 2015). SNPs with missing call rate > 5%, minor allele frequency (MAF) < 5%, or violation of Hardy-Weinberg equilibrium (HWE) with *p*-value < 1e-30, as well as samples with missing call rate > 5%, outliers on heterozygosity or sex mismatches were removed. This final data set included 179 subjects and 289,296 genotyped SNPs. Then we utilized the Sanger Imputation Service for imputation. After the post-imputation QC and the imputation accuracy filter (INFO-score) > 0.80, the final data set included 23,752,992 SNPs.

To describe the population stratification, we performed principal component analysis using SMARTPCA (Patterson et al., 2006) on this pruned dataset of genotyped SNPs (with r2 < 0.20, sliding window of 50 and an increment of 5 SNPs).

**FIGURE 1:**
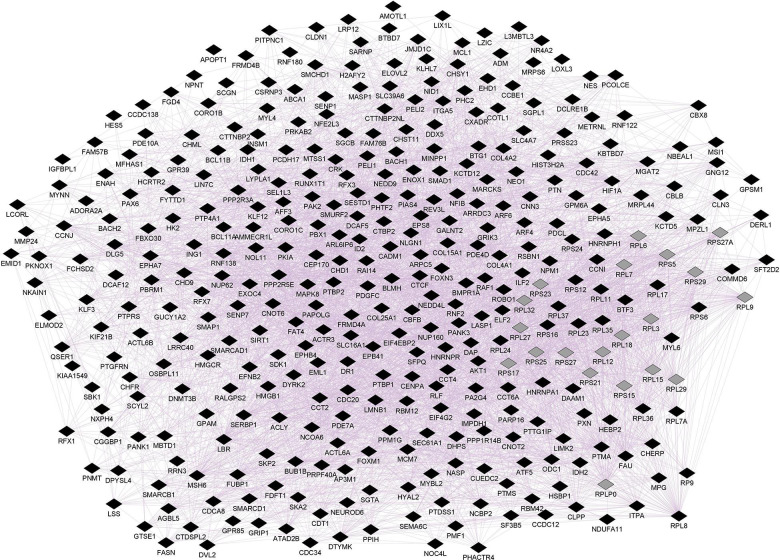
Prefrontal CNR1 gene network using GeneMANIA. Black diamonds indicate query genes, whereas gray diamonds indicate related genes added by GeneMANIA. GeneMANIA converts mRNA expression data from Gene Expression Omnibus (GEO) to functional association networks, connecting co-expressed genes through purple lines. Node sizes represent gene scores, reflecting how often paths that start at a given gene node end up in one of the query genes.

**FIGURE 2:**
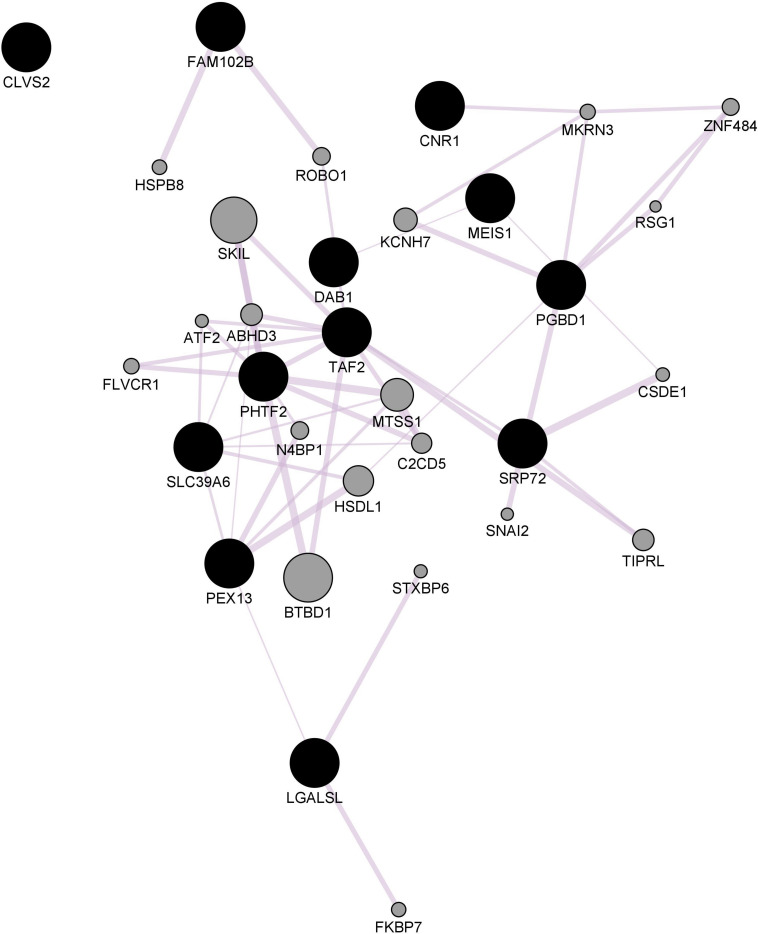
Striatal CNR1 gene network using GeneMANIA. Black circles indicate query genes, whereas gray circles indicate related genes added by GeneMANIA. Node sizes represent gene scores, reflecting how often paths that start at a given gene node end up in one of the query genes.

**FIGURE 3:**
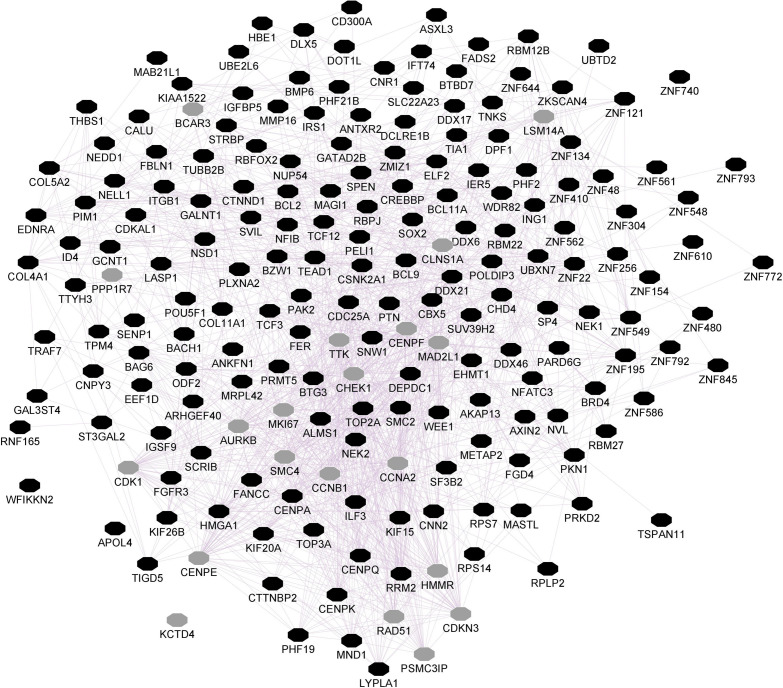
Hippocampal CNR1 gene network using GeneMANIA. Black hexagons indicate query genes, whereas gray hexagons indicate related genes added by GeneMANIA. Node sizes represent gene scores, reflecting how often paths that start at a given gene node end up in one of the query genes.

The *ePRS* was created considering genes co-expressed with the Cannabinoid Receptor (ePRS-CNR1) in the prefrontal cortex (see Figure 1), striatum (see Figure 2), and hippocampus (see Figure 3) according to the protocol previously described by Silveira et al. (2017) and Hari Dass et al. (2019). In summary, the genetic score was created using (a) Genenetwork^1^, (b) Brainspan^2^, and (c) GTEx^3^. In (a), we identified the transcriptional co-expression profiles of CNR1 (4,704 genes co-expressed with CNR1 in mice prefrontal cortex, 1,717 genes co-expressed with CNR1 in mice hippocampus, and 86 genes co-expressed with CNR1 in mice striatum *r* > 0.5) (GeneNetwork). These genes were filtered by selecting those that were overexpressed during fetal/childhood (up to 5 years of age) at 1.5-fold more than adult gene expression in human postmortem samples (Brainspan). The final list included 343 genes for the CNR1 prefrontal gene network, 12 genes for the striatal network, and 175 genes for the hippocampal network. Based on the functional annotation of these genes in the National Center for Biotechnology Information, United States National Library of Medicine^4^ using GRCh37.p13, we gathered all of the existing SNPs from these genes present on our data, merged this list with SNPs that were available on GTEx, and retained the resulting list of SNPs for linkage disequilibrium clumping (*r*2, 0.25). The final lists of SNPs included 8506 independent functional SNPs for CNR1 prefrontal ePRS, 3446 SNPs for the hippocampal ePRS, and 434 SNPs for the striatal network. Based on the children’s genotype data, alleles at a given *cis*-SNP were weighed by the estimated effect of the genotype on gene expression (GTEx in which the effect allele is the alternative allele). Final ePRSs were obtained by summation over all SNPs accounting for the sign of correlation coefficient between the genes and CNR1 gene expression in the different regions. For inclusion in modeling, the CNR1 ePRS scores were standardized. Enrichment analysis of the gene networks was done using MetaCore^®^ (Clarivate Analytics^5^). Cytoscape^®^ software (Shannon, 2003) and GeneMANIA app (Franz et al., 2018) were used to visualize the gene networks. The nodes are the elements of a network, and edges are the connection between these elements, that represent co-expression. Further, CNR1 ePRSs were then categorized into two groups, through a median split to characterize children into low or high ePRS groups.

#### Outcome

Attachment patterns were measured at a mean age of 22 months via the Strange Situation Procedure (SSP), the gold standard assessment for attachment patterns in infancy (Ainsworth et al., 1978). The coding scale was originally designed for children between 12 and 20 months of age but is commonly used up to 24 months (Ainsworth et al., 1978; van IJzendoorn and Kroonenberg, 1988; Solomon and George, 2016). The SSP procedure contains eight brief episodes designed to activate infant’s attachment behaviors by evoking mild levels of stress in children (e.g., seeking proximity to the parent) through a series of mother–child separations and reunions between the infant and mother and interactions between the infant and a ‘stranger’ (a research confederate), where the child’s behaviors were observed through a two-way mirror and video-recorded for coding. A coder (Author Hart) deemed reliable by Alan Sroufe of the Institute of Child Development at the University of Minnesota (ABCD Model) and by Marinus van Ijzendoorn at Cambridge University (Type D) using the Main and Solomon Coding System, coded all SSP videotapes for patterns of attachment using standard categories of secure (B), insecure with subtypes avoidant (A) and resistant (C), and disorganized (D; Ainsworth et al., 1978; Main and Solomon, 1986). To attain a D code, Main’s coding scheme was applied (Main and Stadtman, 1981; Main and Solomon, 1990), which assesses the degree of disorganized behavior in an interpretive way regarding conflict (e.g., aggressive outbursts) and/or disruptive behaviors (e.g., immobilized, disoriented, misdirected, behavior, sudden disordered activities, uninterpretable noises or movements) during the SSP. An expert coder at the Institute for Child Development also re-coded a random 15% of recordings. Cohen’s kappa for inter-rater reliability was 0.73. Due to the relatively small group sample sizes of insecure category subtypes (A and C) and disorganized (D), we dichotomized the sample into two groups, which is common in published research (Lewis-Morrarty et al., 2015; Fresno et al., 2018). The dichotomized groups used in the analyses were comprised of infants classified as secure (B) versus insecure (A, C, and D) and disorganized (D) versus organized (A, B, C).

#### Covariates

Demographic (i.e., maternal age, education, marital status, household income, country of birth; infant birth weight, gestational age, and sex) and descriptive (i.e., depression and social support) variables were considered. Mothers’ perceptions of the quality of their partners’ social support at 3, 6, and 22 months postnatal were assessed via the Social Support Effectiveness Questionnaire (SSEQ). The SSEQ is a 35-item measure that evaluated the type (i.e., emotional/affirmational, informational, instrumental, and negative) and self-perceived effectiveness of the support mothers received from their partner or another support person. Total scores range from 0 to 80, with higher scores indicating more effective support from partners. The internal consistency for this instrument is strong (Cronbach’s alpha = 0.87) when used to distinguish levels of social support for childbearing women (Rini et al., 2006; Stapleton et al., 2011; Giesbrecht et al., 2017). The Edinburgh Postnatal Depression Scale (EPDS) was employed at 3, 6, 12, and 22 months postpartum. On a 10-item self-administered scale, the parent is asked to select the number next to the response closest to how they have felt in the past 7 days. For women, the EPDS has been found to have high sensitivity (83.6%) and specificity (88.3%) for identifying depressive symptoms and the widely accepted cut-off of EPDS ≥ 10, indicating at least probable minor depression (Pop et al., 1992; Matthey et al., 2006). We attempted to employ latent class analysis for both covariates to reduce the data collected at multiple time points (three times for social support, four times for depression). Only the analysis of social support revealed latent classes, categorized as high and low support. As no latent classes were identified for depression, we selected the maximum value on the depression scale over the four measurement time points and employed that value in analyses.

### Statistical Analyses

First, the sample characteristics were analyzed with descriptive summaries, including frequencies, means, and standard deviations as appropriate. Second, univariate logistic regression associations between sample characteristics and attachment security/insecurity and disorganization/organization were examined to identify significant covariates for inclusion in the modeling that follows. Third, logistic regression modeling was employed to examine the main effects of the CARE-Index (sensitivity, controlling, and unresponsiveness) separately (*X* variable) using ePRS for CNR1gene networks in the prefrontal cortex, striatum, and hippocampus (*Z* variable; Model 2) and their interaction terms (Model 3), adjusting for principal components (PCs) for ancestry and sex of the child, along with any identified covariates above. We fitted each model to the data by maximum likelihood and ranked the models by their Akaike Information Criterion (AIC) to control for overfitting (Akaike, 1973). Further, to aid in visualizing the results, we computed the unadjusted predicted probability of attachment pattern for each value of the parental caregiving quality predictors (CARE-Index sensitivity, controlling, and unresponsiveness) considering interaction with ePRS categorized into low (−1SD) and high (+1SD) scores.

#### Analysis of Differential Susceptibility

An additional step was performed in models with a significant interaction term to ensure that any observed differential susceptibility effects were not an artifact of imposing linear model assumptions on non-linear relationships (Model 4). Following the recommendations outlined by Roisman et al. (2012), additional linear regression models, including *X*^2^ and *Z*^∗^*X*^2^ as predictors, were created to verify that neither of these two terms were statistically significant. A *post hoc* analysis for the interaction terms in model 3 included analysis of Proportion of Interaction (PoI; i.e., the proportion of the total area represented in the interaction plots uniquely attributable to differential susceptibility) and Proportion Affected (PA; i.e., the proportion of the population that is differentially affected by the moderator–*Z* variable; Roisman et al., 2012). The regions of significance (RoS) analyses were conducted using a Web-based program developed by Fraley^6^. Further, as per Roisman et al. (2012), evidence for differential susceptibility can be confirmed when the RoS analyses are performed to determine whether the moderator (*Z* variable) and the outcome variable are correlated at the low and high ends of the distribution of the predictor (*X* variable). Results should be considered significant only within a certain range of interest, that is ±2*SD* of the observed predictor variable. Values for the PoI index should be approximately within 0.40 and 0.60, and for the PA index should be close to 0.50 (Roisman et al., 2012; Portella et al., 2020).

## Results

Table 1 presents a descriptive analysis of the study variables. The mean age of mothers was 31.40 (*SD* = 3.90) years. The majority of women were married (98.6%), had attained a university degree or more (69.72%), and had household incomes ≥$70,000 (81.69%). Males made up approximately half of the sample of children (50.7%), and most of the mothers were born in Canada (78.9%). Less than half of children demonstrated a secure (48%) rather than an insecure attachment pattern (52%). Table 2 presents the results of the bivariate analyses of associations between predictors and attachment pattern, revealing that only birth weight significantly predicts disorganization.

**TABLE 1:** Sociodemographic and descriptive characteristics of study participants.

Variables	Frequency	Percentages
Maternal age in years [mean (*SD*)]	31.4 [3.90]	
Gestational age at birth in weeks [mean (*SD*)]	39.34 [1.57]	
Birth weight in kilograms [mean (*SD*)]	3.41 [0.51]	
**Secure attachment**		
Yes	68	47.9%
No	74	52.1%
**Disorganized attachment**		
Yes	17	12.0%
No	125	88.0%
**Sex of child**		
Male	72	50.7%
Female	70	49.3%
**Household income**		
Below $70,000	26	18.3%
70,000 or more	116	81.7%
**Marital status**		
Single	2	1.4%
Married	140	98.6%
**Ethnicity**		
Non-Caucasian	24	16.9%
Caucasian	118	83.1%
**Born in Canada**		
No	30	21.1%
Yes	112	78.9%
**Education level**		
Below degree	43	30.3%
Degree or more	99	69.7%
**Social support (latent class)**		
Class 1 (low social support)	72	50.7%
Class 2 (high social support)	70	49.3%
**Depressive symptoms max value (3, 6, 12, and 22 months)**		
EPDS < 9	102	71.8%
EPDS ≥ 10	40	28.2%

**TABLE 2:** Associations between predictors and attachment pattern.

Variables	Secure	Insecure	OR 95% CI	Not disorganized	Disorganized	OR 95% CI
			*P*-value	[*n* (%)]	[*n* (%)]	*P*-value
Maternal age in years [mean (*SD*)]	31.69 (3.97)	31.13 (3.8)	1.04 (0.95, 1.13) *p* = 0.393	31.43 (3.81)	31.17 (4.66)	0.98 (0.86, 1.12) *p* = 0.769
Gestational age at birth	39.41 (1.24)	39.28 (1.8)	1.05 (0.85, 1.29)	39.41 (1.52)	38.87 (1.88)	0.82 (0.62, 1.10)
Birth in weeks [mean (*SD*)]			*p* = 0.639			*p* = 0.185
Birth weight in kg [mean (*SD*)]	3.48 (0.48)	3.34 (0.54)	1.74 (1.89, 3.36) *p* = 0.101	3.45 (0.49)	3.07 (0.57)	0.23 (0.08, 0.65) ***p*** = **0.005**
ePRS CNR1	−0.15 (0.90)	0.14 (1.06)	0.74 (0.53, 1.04)	−0.07 (0.97)	0.55 (1.08)	1.97 (1.13, 3.43)
			*p* = 0.087			***p*** = **0.017**
Maternal sensitivity	5.19 (1.75)	5.33 (2.27)	0.96 (0.69, 1.34)	5.32 (1.89)	5.0 (3.01)	0.65 (0.38, 1.15)
			*p* = 0.806			*p* = 0.139
Maternal controlling	2.70 (3.68)	2.61 (3.46)	0.97 (0.70, 1.35)	2.52 (3.47)	3.75 (4.02)	1.42 (0.89, 2.25)
			*p* = 0.856			*p* = 0.139
Maternal unresponsiveness	6.07 (3.45)	6.05 (3.71)	1.05 (0.75, 1.46)	6.14 (3.58)	5.25 (3.49)	0.84 (0.51,1.39)
			*p* = 0.783			*p* = 0.510
**Sex**						
Male	31 (45.59)	41 (55.41)	1.48 (0.76, 2.87)	64 (51.20)	8 (47.06)	1.16 (0.42, 3.20)
Female	37 (54.41)	33 (44.59)	*p* = 0.243	61 (48.80)	9 (52.94)	*p* = 0.748
**Household income**						
Below $70,000	12 (17.65)	14 (18.92)	1.09 (0.46, 2.55)	21 (16.80)	5 (29.41)	0.48 (0.15, 1.51)
70,000 or more	56 (82.35)	60 (81.08)	*p* = 0.845	104 (83.20)	12 (70.59)	*p* = 0.207
**Born in Canada**						
No	15 (22.06)	15 (20.27)	0.89 (0.40, 2.01)	25 (20.0)	5 (29.41)	0.59 (0.19, 1.84)
Yes	53 (77.94)	59 (79.73)	*p* = 0.794	100 (80.0)	12 (70.59)	*p* = 0.372
**Education level**						
Below university degree	22 (32.35)	21 (28.38)	0.83 (0.40, 1.69)	38 (30.40)	5 (29.41)	1.03 (0.34, 3.14)
University degree or more	46 (67.65)	53 (71.62)	*p* = 0.607	87 (69.60)	12 (70.59)	*p* = 0.934
**Maternal depression (max value)**						
Not depressed	47 (69.12)	54 (73.97)	1.27 (0.61, 2.64)	93 (73.81)	9 (56.25)	2.19 (0.76, 6.35)
Depressed	21 (30.88)	19 (26.03)	*P* = 0.523	33 (26.19)	7 (43.75)	*p* = 0.148
**Social support (latent class)**						
Low social support	36 (53.73)	36 (48.65)	0.82 (0.42, 1.58)	66 (52.80)	6 (35.29)	2.05 (0.71, 5.89)
High social support	31 (46.27)	38 (51.35)	*p* = 0.547	59 (47.20)	11 (64.71)	*p* = 0.176

*Bolded indicates a p value of less than 0.05.*

### ePRS CNR1 Prefrontal Cortex

Hypothesis 1. We hypothesized that higher maternal sensitivity would interact with CNR1 ePRS in the prefrontal cortex in predicting a higher probability of secure attachment and reduced probability of disorganization, controlling for covariates. With respect to the probability of attachment security or disorganization, logistic regression revealed no significant associations in any model (results not shown).

Hypothesis 2. We hypothesized that higher maternal controlling would interact with CNR1 ePRS in the prefrontal cortex in predicting a reduced probability of secure attachment and a higher probability of disorganization. With respect to both attachment security and disorganization, logistic regression revealed no significant interactions (results not shown).

Hypothesis 3. We hypothesized that higher maternal unresponsiveness would interact with CNR1 ePRS in the prefrontal cortex to predict a reduced probability of secure attachment and a higher probability of disorganization. With respect to both attachment security and disorganization, logistic regression revealed no significant interactions (results not shown).

### ePRS CNR1 Striatum

**FIGURE 4:**
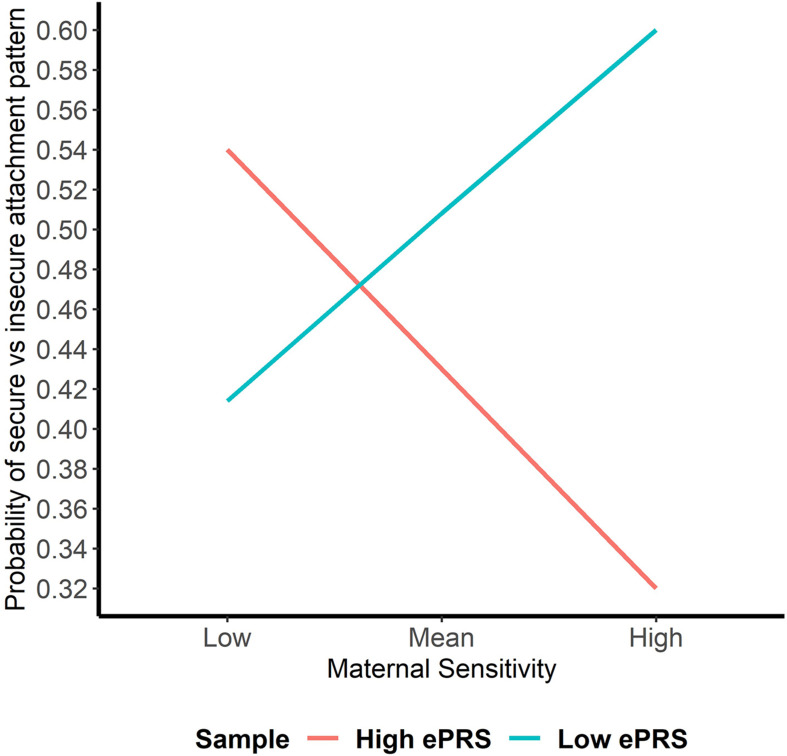
Interaction between Striatal Gene Network and Maternal Sensitivity in Predicting Attachment. Shows that higher maternal sensitivity and a low CNR1 ePRS in the striatum predicts a higher probability of secure attachment. Higher maternal sensitivity and a high CNR1 ePRS in the striatum predicts a lower probability of secure attachment.

Hypothesis 1. We hypothesized that higher maternal sensitivity would interact with CNR1 ePRS in the striatum in predicting a higher probability of secure attachment and reduced probability of disorganization, controlling for covariates. We observed a significant interaction between striatum CNR1 ePRS and maternal sensitivity in predicting the probability of attachment security in the fully adjusted model 3 (see Table 3). This model complied with a differential susceptibility assessment, given that the crossover point was within the limits of the *X* variable (maternal sensitivity) and indices were near expected values (PoI = 0.67, PA = 0.63). See Figure 4 for the graphed associations. With respect to the probability of disorganization, logistic regression revealed no significant associations in any model (results not shown).

**TABLE 3:** Associations among maternal sensitivity, striatal gene network for CNR1ePRS, covariates, and secure vs. insecure attachment pattern.

Variables	Model 1 Adjusted OR (95% CI)	Model 2 Adjusted OR (95% CI)	Model 3 Adjusted OR (95% CI)	Model 4 Adjusted OR (95% CI)
Maternal sensitivity	0.93 (0.67, 1.31) *p* = 0.693	–	0.93 (0.64, 1.34) *p* = 0.929	4.8- (0.57, 40.31) *p* = 0.148
Maternal sensitivity^2^	–	–	–	0.92 (0.83, 1.02) *p* = 0.123
Striatal_ePRS	–	0.90 (0.64,1.26) *p* = 0.542	0.86 (0.59, 1.23) *p* = 0.414	1.05 (0.65,1.70) *p* = 0.829
Maternal sensitivity × hippocampal ePRS	–	–	**0.64 (0.43, 0.96) *p*** = **0.031**	**0.54 (0.32, 0.91) *p*** = **0.019**
Maternal sensitivity^2^ × striatal ePRS	–	–	**–**	0.71 (0.44, 1.13) *p* = 0.152
Female	1.51 (0.77, 2.94) *p* = 0.227	0.64 (0.33, 1.25) *p* = 0.193	0.59 (0.29, 1.19) *p* = 0.144	1.64 (0.81, 3.34) *p* = 0.167
PC1	–	0.10 (0.0, 18.51) *p* = 0.388	0.10 (0.00, 18.52) *p* = 0.387	0.06 (0.00, 12.76) *p* = 0.303
PC2	–	165.81 (0.00, 3.15E + 9) *p* = 0.410	244.85 (0.00, 5.05E + 9) *p* = 0.378	168.38 (0.00, inf) *p* = 0.417
PC3	–	0.01 (0.00, 19.42) *p* = 0.250	0.01 (0.00, 11.31) *p* = 0.191	0.0 (0.00, 6.75) *p* = 0.141
AIC	201.07	204.90	203.03	200.39
PoI	–	–	0.67	–
Crossover point		–	−0.34	–
PA index	–	–	0.63	–

*CI, confidence interval; OR, odds ratio. PC 1, 2, 3, principal component for ancestry; AIC, Akaike Information Criterion; PoI, proportion of interaction; PA, proportion affected; inf, very large CI and OR; bold refers to p < 0.05. Logistic regression modeling for main effects of the sensitivity (X variable; model 1) using ePRS for CNR1 gene networks in the striatum (Z variable; model 2) and their interaction terms (Model 3), adjusting for principal components (PCs) for ancestry and sex of the child. Model fit statistics (AIC; model) confirmed that Model 3 was optimal. Bolded indicates a p value of less than 0.05.*

Hypothesis 2. We hypothesized that higher maternal controlling would interact with CNR1 ePRS in the striatum in predicting a reduced probability of secure attachment and a higher probability of disorganization. With respect to both attachment security and disorganization, logistic regression revealed no significant interactions (results not shown).

Hypothesis 3. We hypothesized that higher maternal unresponsiveness would interact with CNR1 ePRS in the striatum to predict a reduced probability of secure attachment and a higher probability of disorganization. With respect to both attachment security and disorganization, logistic regression revealed no significant interactions (results not shown).

### ePRS CNR1 Hippocampus

Hypothesis 1. We hypothesized that higher maternal sensitivity would interact with CNR1 ePRS in the hippocampus in predicting a higher probability of secure attachment and reduced probability of disorganization, controlling for covariates. With respect to both attachment security and disorganization, logistic regression revealed no significant interactions (results not shown).

**FIGURE 5:**
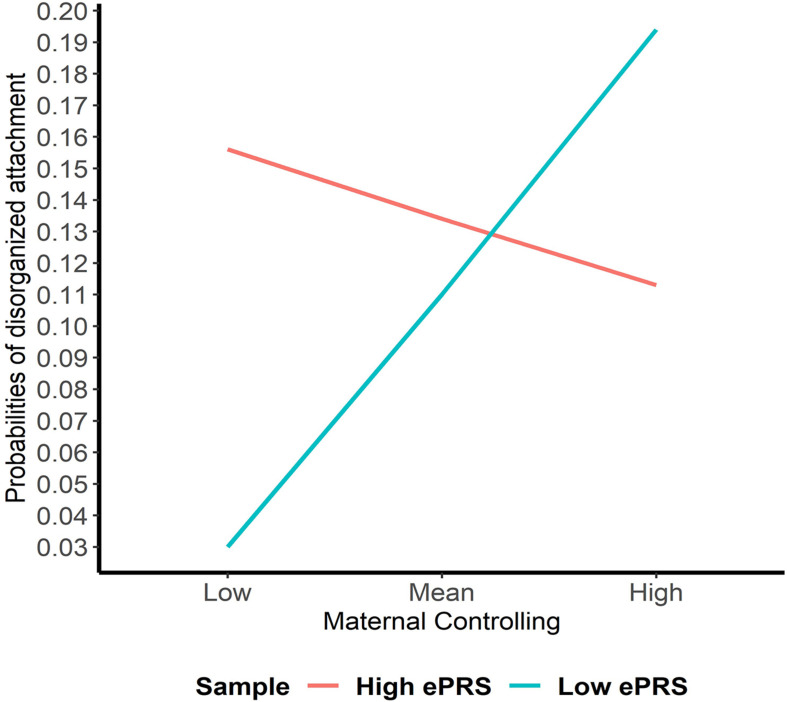
Interaction between Hippocampal Gene Network and Maternal Controlling in Disorganized Attachment. Shows that higher maternal controlling and a high CNR1 ePRS in the hippocampus predicts a lower probability of disorganization. Higher maternal controlling and a low CNR1 ePRS in the hippocampus predicts a higher probability of disorganization.

Hypothesis 2. We hypothesized that higher maternal controlling would interact with CNR1 ePRS in the hippocampus to predict a higher probability of insecure attachment and a higher probability of disorganization, controlling for covariates. With respect to the probability of attachment security (results not shown), logistic regression revealed no significant associations in any model. However, we observed a significant interaction between hippocampal CNR1 ePRS and maternal controlling behavior in predicting the probability of disorganization in the fully adjusted model 3 (see Table 4). This model did not comply with the criteria for differential susceptibility, given that the cross over point was not near the midpoint of *X* or even within the limits of the *X* variable (maternal controlling) and indices were outside of expected values (PoI = 0.16, PA = 0.22), suggesting that this interaction is more indicative of diathesis-stress. See Figure 5 for the graphed associations.

**TABLE 4:** Associations among maternal controlling, hippocampal gene network for ePRS, covariates, and disorganized versus organized attachment pattern.

Variables	Model 1 Adjusted OR (95% CI)	Model 2 Adjusted OR (95% CI)	Model 3 Adjusted OR (95% CI)	Model 4 Adjusted OR (95% CI)
Maternal controlling	1.35 (0.83, 2.21) *p* = 0.221	-	1.58 (0.89,2.81) *p* = 0.112	1.13 (0.61, 2.11) *p* = 0.691
Maternal controlling^2^				1.0 (0.94, 1.06) *p* = 0.973
Hippocampal ePRS	–	1.44 (0.79, 2.59) *p* = 0.229	1.79 (0.92, 347) *p* = 0.083	1.21 (0.45, 3.21) *p* = 0.700
Maternal controlling × hippocampal ePRS	–	–	**0.47 (0.25, 0.89) *p*** = **0.021**	**0.29 (0.10., 0.86) *p*** = **0.026**
Maternal Controlling^2^ × Hippocampal ePRS	–	–	–	1.65 (0.67, 4.08) *p* = 0.270
Female	1.14 (0.39, 3.30) *p* = 0.804	1.23 (0.40, 3.73) *p* = 0.715	0.92 (0.28, 2.95) *p* = 0.883	1.03 (0.31, 3.38) *p* = 0.962
Birth weight (kgs)	**0.25 (0.09, 0.70) *p* = 0.008**	**0.24 (0.08, 0.77) *p* = 0.016**	**0.20 (0.06, 0.72) *p*** = **0.014**	**0.22 (0.06, 0.77) *p* = 0.018**
PC1	–	0.04 (0.00, 149.96) *p* = 0.436	1.05 (0.00, 224.54) *p* = 0.438	0.02 (0.00, 184.59) *p* = 0.401
PC2	–	Inf (0.06, inf) *p* = 0.109	**Inf (0.72, inf) *p* = 0.05**	**Inf (2.17, inf) *p* = 0.039**
PC3	–	2.14 (0.01, inf) *p* = 0.844	1.05 (0.00, inf) *p* = 0.991	1.19 (0.00, inf) *p* = 0.963
AIC	102.28	104.63	101.15	103.85
PoI	–	–	0.16	–
Crossover point	–	–	0.78	–
PA index	–	–	0.22	–

*CI, confidence interval; OR, odds ratio. PC 1, 2, 3, principal component for ancestry; AIC, Akaike Information Criterion; PoI, proportion of interaction; PA, proportion affected; inf, very large CI and OR; bold refers to p < 0.05. Logistic regression modeling for main effects of the controlling (X variable; Model 1) using ePRS for CNR1 gene networks in the hippocampus (Z variable; Model 2) and their interaction terms (Model 3), adjusting for principal components (PCs) for ancestry and sex of the child. Model fit statistics (AIC) confirmed that Model 3 was optimal. Bolded indicates a p value of less than 0.05.*

**FIGURE 6:**
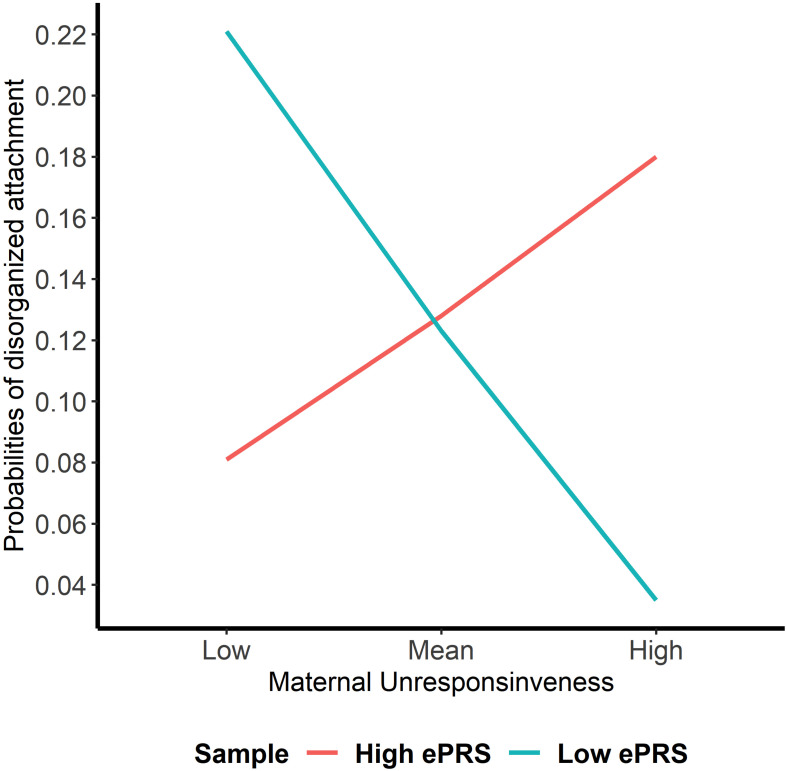
Interaction between Hippocampal Gene Network and Maternal Unresponsiveness in Disorganized Attachment. Shows that high maternal unresponsiveness and a low CNR1 ePRS in the hippocampus predict a lower probability of disorganization. Higher maternal unresponsiveness and a high CNR1 ePRS in the hippocampus predict a higher probability of disorganization.

Hypothesis 3. We hypothesized that higher maternal unresponsiveness would interact with CNR1 ePRS in the hippocampus in predicting a higher probability of insecure attachment and higher probability of disorganization, controlling for covariates. With respect to the probability of attachment security, logistic regression revealed no significant associations in any model (results not shown). We observed a significant interaction between hippocampal CNR1 ePRS and maternal unresponsiveness in predicting the probability of disorganization in the fully adjusted model 3 (see Table 5). See Figure 6 for the graphed association. This model did not comply with the criteria for the differential susceptibility model, given that the cross over point was not near the midpoint of *X* or even within the limits of the *X* Variable (maternal unresponsiveness) and indices were outside of expected values (PoI = 0.73, PA = 0.68), suggesting that this interaction is more indicative of diathesis–stress.

**TABLE 5:** Associations among maternal unresponsiveness, hippocampal gene network for ePRS, covariates, and disorganized versus organized attachment pattern.

Variables	Model 1 Adjusted OR (95% CI)	Model 2 Adjusted OR (95% CI)	Model 3 Adjusted OR (95% CI)	Model 4 Adjusted OR (95% CI)
Maternal unresponsiveness	0.88 (0.51, 1.49) *p* = 0.629	–	0.69 (0.36, 1.32) *p* = 0.261	4.45 (0.41, 47.88) *p* = 0.218
Maternal unresponsiveness^2^	–	–	–	0.94 (0.88, 1.01) *p* = 0.088
Hippocampal ePRS	–	1.44 (0.79, 2.59) *p* = 0.229	1.57 (0.81, 3.03) *p* = 0.182	1.17 (0.51, 2.68) *p* = 0.700
Maternal unresponsiveness × hippocampal ePRS	–	–	**2.56 (1.29, 5.08) *p*** = **0.007**	**4.36 (1.78, 10.64) *p* = 0.001**
Maternal unresponsiveness^2^ × hippocampal ePRS	–	–	–	2.08 (0.95,4.52) *p* = 0.065
Female	1.12 (0.39, 3.19) *p* = 0.843	1.23 (0.40, 3.73) *p* = 0.715	0.96 (0.30, 3.08) *p* = 0.949	0.98 (0.28, 3.40) *p* = 0.983
Birth weight (kgs)	0.24 (0.08, 0.67) *p* = 0.006	0.24 (0.08, 0.77) *p* = 0.016	**0.16 (0.04, 0.61) *p* = 0.007**	**0.13 (0.03, 0.53) *p* = 0.004**
PC1	–	0.04 (0.00, 149.96) *p* = 0.436	0.05 (0.00, 376.37) *p* = 0.509	(0.00, 196.55) *p* = 0.378
PC2	–	Inf (0.06, inf) *p* = 0.109	Inf (1.99, inf) *p* = 0.119	**Inf (361.76, inf) *p* = 0.010**
PC3	–	2.14 (0.01, inf) *p* = 0.844	0.74 (0.001, 2.14 E + 7) *p* = 0.940	0.05 (0.00, 356.35) *p* = 0.519
AIC	103.50	104.64	99.71	98.04
PoI	–	–	0.73	–
Crossover point	–	–	−0.48	–
PA index	–	–	−0.68	–

*CI, confidence interval; OR, odds ratio. PC 1, 2, 3, principal component for ancestry; AIC, Akaike Information Criterion; PoI, proportion of interaction; PA, proportion affected; inf, very large CI and OR; bold refers to p < 0.05. Logistic regression modeling for main effects of the unresponsiveness (X variable; Model 1) using ePRS for CNR1 gene networks in the hippocampus (Z variable; Model 2) and their interaction terms (Model 3), adjusting for principal components (PCs) for ancestry and sex of the child. Model fit statistics (AIC) confirmed that Model 3 was optimal. Bolded indicates a p value of less than 0.05.*

### Gene Network Analysis

Enrichment analysis demonstrated the prefrontal CNR1 gene network is enriched for gene ontology terms related to nervous system development (FDR q = 7.493e-16), regulation of neuron differentiation (FDR q = 1.958e-13), and neurogenesis (FDR q = 2.514e-13). The hippocampal network is enriched for gene ontology terms related to regulation of transcription (FDR q = 1.757e-21) and regulation of metabolic processes (FDR q = 1.022e-21). The striatal network is enriched for GO terms related to transcription initiation (FDR q = 7.619e-9), histone acetylation (FDR q = 2.124e-5), and the cannabinoid signaling pathway (FDR q = 4.786e-4).

## Discussion

This study set out to analyze if parental caregiving qualities (i.e., sensitivity, controlling, and unresponsiveness) interacted with the ePRS for the CNR1 gene networks in the prefrontal cortex, striatum, and hippocampus in predicting the probability of secure or disorganized attachment patterns. We hypothesized that higher sensitivity, lower controlling, and lower unresponsiveness would interact with ePRS for CNR1 in these three brain regions in predicting a higher probability of secure attachment and reduced probability of disorganization. Results for the prefrontal cortex failed to reject the null hypotheses for interaction effects between sensitivity, unresponsiveness, and controlling with CNR1 ePRS on either security of attachment or disorganization. Within the striatum, we observed a significant interaction between maternal sensitivity and CNR1 ePRS in predicting attachment security. We observed that higher maternal sensitivity and a low CNR1 ePRS in the striatum predicted a higher probability of secure attachment. The opposite is true for high CNR1 ePRS; higher maternal sensitivity and a high CNR1 ePRS in the striatum predicts a lower probability of secure attachment. Within the hippocampus, we observed a significant interaction between both unresponsiveness and controlling with the CNR1 ePRS in predicting disorganization. Higher maternal controlling and a higher CNR1 ePRS in the hippocampus predicted a lower probability of disorganization, and higher maternal controlling with a lower CNR1 ePRS predicted a higher probability of disorganization. Finally, we observed that high maternal unresponsiveness coupled with a low CNR1 ePRS in the hippocampus predicted a lower probability of disorganization and higher maternal unresponsiveness with a high CNR1 ePRS predicted a higher probability of disorganization.

In summary, low CNR1 ePRS in the striatum, a region of the brain involved in the reward motivation system, predicted a greater likelihood of secure attachment in the context of more optimal parental caregiving (i.e., greater sensitivity). Within the hippocampus, a region of the brain known for its involvement in spatial and emotional memory, suboptimal parental caregiving (i.e., greater degrees of controlling and unresponsive parental behavior) predicted a decreased likelihood of disorganized attachment with a high CNR1 ePRS with respect to maternal controlling and a low CNR1 ePRS with respect to maternal unresponsiveness. Our findings offer support for the genetic differential susceptibility to the quality of maternal sensitivity within the context of the CNR1 ePRS in the striatum, as suggested by Belsky (1997), who theorized that children may differ in their receptiveness to parenting influences. However, in the case of the significant interactions between hippocampal CNR1 ePRS and maternal unresponsiveness and controlling in predicting the probability of disorganization, the analyses carried out to confirm differential susceptibility were more suggestive of the diathesis-stress model. The diathesis-stress model suggests that poor developmental experiences (e.g., low-quality parenting) will have the greatest impact on the development of individuals who carry vulnerability factors (e.g., genetic polymorphisms), which are latent diatheses that result in maladaptation when “turned on” by poor environmental experiences (Garmezy et al., 1984; Roisman et al., 2012). These findings are consistent when examining the role that genetics may play in how children form attachments, as other studies have observed that parenting particularly affected children with various polymorphisms of genes that regulate the DA system (i.e., DAT1 9- and 10-repeat and Dopamine Receptor D4 7-repeat) and reward sensitivity (Bakermans-Kranenburg et al., 2008; Bosmans et al., 2020). Our findings further support the notion that multiple genes may make a child more or less susceptible to their caregiving environment (Belsky and Beaver, 2011; Roisman et al., 2012), but in a manner consistent with either differential susceptibility or diathesis-stress, given the brain region under study.

Attachment is a relationship between infants and their caregivers, representing a brain-based biological evolutionary system promoting infant survival (Ainsworth et al., 1978; Chisholm, 1996). In attachment pattern formation, activation is observed among the neurobiological systems underpinning affiliation, reward, and stress management (Ulmer-Yaniv et al., 2016). These observations are likely a result of the intrinsic motivational value that combines the immediate hedonic responses in developing bonds with approach motivation, goal-directed behavior, and learning (Berridge and Robinson, 1998). Our findings related to the CNR1 gene network in the prefrontal cortex, striatum, and hippocampus corroborates the associations between the genetic variations within the ECS and attachment pattern formations. When primary caregivers/parents provide a supportive environment, infants develop a sense of security, making them feel safe, secure, and protected (Bowlby, 1982; Benoit, 2004; Solomon and George, 2016). In contrast, evidence suggests that disorganized attachment is predicted by sub-optimal parenting and can lead to child behavioral and lifespan mental health problems (Main and Solomon, 1990; Lakatos et al., 2000, 2002; Sroufe, 2005; Puig et al., 2013).

The CNR1 gene networks within the prefrontal cortex, striatum, and hippocampus were chosen to be examined within the context of differential susceptibility, yet findings also pointed to the diathesis-stress model. CNR1 gene has been identified through extensive research as having polymorphisms associated with different observable outcomes (e.g., externalizing behavior and self-regulation) in response to differences in parenting/caregiving qualities (Belsky and Pluess, 2009b; Belsky and Beaver, 2011). In addition, these gene networks were examined within these brain regions because of the existence of convergent projections from the cortex to the striatum, along with hippocampal and amygdala-striatal projections, that places the striatum as a central entry port for processing emotional/motivational information in supporting the development of human attachments (Feldman, 2017). While several studies have focused on the effects of specific variations of these genes in relation to behavior and self-regulation (Belsky and Pluess, 2009b; Belsky and Beaver, 2011; Letourneau et al., 2019), this is the first study to our knowledge that not only examines the associations between these genes and attachment patterns but also utilizes ePRS to predict the probability of disorganized attachment patterns. Our findings suggest that it is important to consider both the ePRS and the brain region when looking at a child’s susceptibility to their caregiving environment and provide promise for examining these gene networks in other regions of the brain or other gene networks where a candidate gene approach has been associated with varying attachment patterns and differential susceptibility or diathesis-stress [e.g., dopamine receptor D4 gene (DRD4) and a disorganized attachment pattern; Lakatos et al., 2000; Bakermans-Kranenburg and van Ijzendoorn, 2016].

Attachment theory provides a framework that explains the influence of early social experiences on normal and problematic development (Lakatos et al., 2000). Even in the case of adopted children who are not biologically related to their parents, it was found that early mother–infant interactions and attachment patterns predicted later social-emotional and cognitive development (Stams et al., 2002). Disorganized infant-parent attachment has become an area of significant interest to researchers and clinicians due to its clear associations with lifespan developmental and psychological disorders (Newman et al., 2015). We have demonstrated that variations among the CNR1 gene networks in the various brain regions (i.e., prefrontal cortex, striatum, and hippocampus) demonstrated different findings in predicting secure and disorganized attachment (De Wolff and van Ijzendoorn, 1997; van Ijzendoorn et al., 1999; Madigan et al., 2006; Crittenden, 2010; Cyr et al., 2010; Leerkes, 2011; Bailey et al., 2017). Understanding genetic factors that may affect the security of an infant’s attachment with the mother may help identify those at risk for attachment disorganization by adding predictive possibility (Bakermans-Kranenburg and van Ijzendoorn, 2007). Failure to consider a child’s genotype and differential susceptibility (or diathesis-stress) to experiences (e.g., caregiver sensitivity, responsiveness, and controlling) may pose a barrier to understanding the broader set of predictors of secure attachment pattern and undermine interventions aimed at changing a child’s socioenvironmental exposures.

### Limitations and Strengths

This study has many strengths, including the prospective design and observational assessments of maternal-child relationship quality (i.e., sensitivity) and attachment patterns; however, there are several limitations to note. First, the sample that we employed for this secondary data analysis is highly educated (69.72% of mothers having a university degree) as compared with the provincial (28.2%) and national (28.5%) averages, which may limit generalizability (Letourneau et al., 2019; Statistics Canada, 2020). Further, the majority of women were married (98.6%) and had household income ≥$70,000 (81.69%). Finally, parity or the presence of siblings for each child was not factored into the analysis, potentially affecting the maternal perception of infant cues, thereby affecting maternal sensitivity (Rutherford et al., 2017). In addition, only “maternal” caregiving quality was assessed; however, rather than seeking to reinforce gender stereotypes, we recognize that primary caregivers may be mothers, fathers, or others. We also recognize that in Canada (Findlay and Kohen, 2012) and in our study (Kaplan et al., 2014), the majority of primary caregivers of infants are mothers.

## Conclusion

To the best of our knowledge, this is the first study that examines the interaction between maternal parental caregiving qualities (i.e., sensitivity, controlling, and unresponsiveness) and children’s ePRS for the CNR1 gene networks in the prefrontal cortex, striatum, and hippocampus in predicting the probability of secure and disorganized attachment patterns in young children. This research provides a foundation to explore genetic susceptibilities to varying caregiving environments in predicting attachment patterns and other outcomes. This research also provides a starting point for exploring other gene networks and influences on children’s differential susceptibility to their environments. Promoting secure attachment patterns is a public health goal, as it is associated with lifelong health and a reduced likelihood of all-cause morbidity, chronic inflammation, coronary artery disease, and an array of mental disorders. Further research in the area may allow practitioners to target interventions to support those most at risk for insecure or disorganized attachment, thereby reducing the risk for negative life-long sequelae.

## Data Availability Statement

The data generated for this study is subject to the following licenses/restrictions: Privacy and Confidentiality of Participants. Requests to access these datasets should be directed to NL, nicole.letourneau@ucalgary.ca.

## Ethics Statement

The studies involving human participants were reviewed and approved by University of Calgary Conjoint Health Research Ethics Board. Written informed consent to participate in this study was provided by the participants’ legal guardian/next of kin.

## Author Contributions

AP-D contributed to data analysis and graphing of genes, wrote and edited the drafts. NL devised the project, conceived the research questions, oversaw data collection, analysis and graphing of figures, and organized, wrote, and edited the drafts. PS contributed by generating the co-expression gene networks, genotyping data QC and polygenic scores calculation. HN conducted the data analysis, described the analysis and results, and prepared all tables. AK and SD reviewed drafts and offered substantive guidance. MH collected data essential to this project and described the measurement. GG contributed to data collection, reviewed drafts, and offered substantive guidance. All authors contributed to the article and approved the submitted version.

## Conflict of Interest

The authors declare that the research was conducted in the absence of any commercial or financial relationships that could be construed as a potential conflict of interest.

## Publisher’s Note

All claims expressed in this article are solely those of the authors and do not necessarily represent those of their affiliated organizations, or those of the publisher, the editors and the reviewers. Any product that may be evaluated in this article, or claim that may be made by its manufacturer, is not guaranteed or endorsed by the publisher.

## Footnotes

1
http://genenetwork.org

2
http://www.brainspan.org/rnaseq/search/index.html

3
https://www.gtexportal.org/home/

4
https://www.ncbi.nlm.nih.gov/variation/view/

5
https://portal.genego.com/

6
http://www.yourpersonality.net/interaction
